# Effects of cerebellar transcranial direct current stimulation on improving post-stroke upper extremity motor function: a protocol for a randomized controlled clinical trial

**DOI:** 10.3389/fneur.2025.1670721

**Published:** 2025-11-18

**Authors:** Ziqiao Huang, Jiahao Lao, Zhibin Chen, Yuxuan Wu, Bo Tang, Wei Liao, Wanqi Jiang, Junjie Liang, Zirui Luo, Haian Mao

**Affiliations:** 1 The Fifth Affiliated Hospital of Guangzhou Medical University, Guangzhou, China; 2 Department of Rehabilitation, The Fifth Affiliated Hospital of Guangzhou Medical University, Guangzhou, China

**Keywords:** stroke, upper extremity motor function, cerebellum, transcranial direct current stimulation, functional near-infrared spectroscopy

## Abstract

**Background:**

Upper extremity motor impairment is a prevalent and disabling consequence of stroke. While conventional rehabilitation improves function, recovery often plateaus. Cerebellar transcranial direct current stimulation (c-tDCS) presents a promising neuromodulatory adjunct by targeting cerebellar involvement in motor coordination, timing, and learning. However, robust evidence from well-designed randomized controlled trials (RCTs) is needed to establish its efficacy in enhancing post-stroke upper limb recovery.

**Objective:**

This RCT protocol aims to evaluate the efficacy of anodal c-tDCS applied sequentially with conventional upper limb rehabilitation (CULR), compared to sham stimulation plus the same rehabilitation, on improving motor function of the paretic upper extremity in subacute/chronic stroke patients.

**Methods:**

A double-blind, randomized, sham-controlled trial will be conducted. Fourty-eight participants with unilateral stroke and moderate to severe upper limb motor impairment will be randomized to either active or sham group. Anodal tDCS will be conducted to the ipsilesional cerebellar hemisphere in the active group, while sham delivery will be performed in the sham group. Both groups receive CULR after each c-tDCS session. Multimodal assessments will be administered pre- and post-intervention, comprising: Fugl-Meyer assessment upper extremity (FMA-UE) for motor impairment quantification, functional near-infrared spectroscopy (fNIRS) capturing resting-state and c-tDCS-induced cortical hemodynamic responses and transcranial magnetic stimulation-derived motor evoked potentials (TMS-MEPs) evaluating cortical excitability.

**Conclusion:**

This rigorously designed RCT will provide high-level evidence on the therapeutic potential of c-tDCS as an adjunct to rehabilitation for improving upper limb motor function post-stroke. Findings will inform clinical practice regarding novel neuromodulation strategies to augment recovery.

**Clinical trial registration:**

https://www.chictr.org.cn, identifier ChiCTR2500101094.

## Introduction

Stroke represents the leading cause of adult disability worldwide, imposing substantial socioeconomic burdens (1). With approximately 14 million incident strokes annually, up to 75% of survivors experience chronic disabilities (2). Upper extremity (UE) motor impairment persists as a particularly debilitating sequela, significantly compromising activities of daily living (ADL) and quality of life (3).

Post-stroke UE dysfunction stems from complex pathophysiological mechanisms, including imbalanced interhemispheric inhibition, characterized by contralesional M1 hyperexcitability suppressing ipsilesional cortex via transcallosal pathways, and disrupted cerebello-thalamo-cortical (CTC) integration (4, 5). A critical manifestation of CTC disruption is crossed cerebellar diaschisis (CCD), a biomarker associated with poorer motor recovery prognoses (6). These neural network dysfunctions collectively constrain neuroplasticity and limit functional recovery.

While conventional neurorehabilitation (e.g., task-oriented training, neuromuscular electrical stimulation, mirror therapy, robot-assisted therapy or brain-computer interface) yields partial improvements in gross motor function, efficacy for restoring fine motor control (precision grasp, bimanual coordination) and therapeutic sustainability remains limited (7–10). Non-invasive brain stimulation (NIBS), particularly transcranial direct current stimulation (tDCS), offers promise by modulating cortical excitability and enhancing neuroplasticity (11). However, optimal stimulation targets and polarities for UE recovery, especially concerning fine motor function, are inadequately characterized, and the underlying neural mechanisms require elucidation through multimodal assessment.

The cerebellum is integral to motor coordination, timing, and error correction via reciprocal connections with cortical motor areas. Cerebellar tDCS (c-tDCS) presents a compelling neuromodulatory tool. Critically, c-tDCS can modulate bilateral M1 excitability – potentially reducing pathological contralesional hyperexcitability while facilitating ipsilesional activity – thereby promoting restoration of interhemispheric balance (12). This mechanism is especially relevant given the frequent disruption of corticocerebellar pathways post-stroke.

In designing this trial, we selected anodal tDCS applied to the ipsilesional cerebellum over cathodal stimulation of the contralesional cerebellum based on a multi-faceted rationale. Firstly, the stroke lesion and its associated white matter damage likely compromise the cerebello-thalamo-cortical pathway within the affected hemisphere. In contrast, the pathway originating from the contralesional cerebellum remains relatively intact. Therefore, taking advantage of the ipsilesional cerebellum with this intact CTC pathway represents a more safe strategy for promoting recovery. Secondly, emerging evidence suggests that unilateral anodal c-tDCS can exert bidirectional effects on the bilateral motor cortices. Specifically, a study by Shoaib et al. (12) demonstrated in healthy adults that anodal stimulation of the right cerebellum significantly increased cortical activity (reflected by oxyhemoglobin concentration) in the ipsilateral right M1 while simultaneously decreasing activity in the contralateral left M1. This pattern of simultaneous ipsilateral facilitation and contralateral inhibition is ideally suited to counteract the pathological interhemispheric imbalance post-stroke. Thirdly, the rationale for inhibiting the contralesional cerebellum via cathodal tDCS is theoretically less sound. The phenomenon of CCD already indicates a state of functional depression and metabolic hypoactivity in the cerebellar hemisphere contralateral to the cerebral lesion. Applying further inhibitory stimulation to a potentially already suppressed cerebellum may not be physiologically justified, and the effects of cerebellar cathodal tDCS are generally less predictable.

Therefore, this randomized controlled trial aims to evaluate the clinical efficacy of anodal c-tDCS applied to the ipsilesional cerebellar hemisphere, combined with conventional upper limb rehabilitation (CULR), versus sham c-tDCS with CULR, for improving UE motor function in subacute/chronic stroke patients. The primary outcome is the Fugl-Meyer Assessment for the UE (FMA-UE). Furthermore, we aim to elucidate the neurophysiological mechanisms underlying c-tDCS using fNIRS to assess cortical hemodynamic activity pre-, intra-, and post-stimulation. Last but not least, we aim to employ transcranial magnetic stimulation to quantify corticospinal excitability via motor evoked potentials (MEPs) from bilateral M1.

### Hypotheses

We hypothesize that: (1) Participants receiving active anodal c-tDCS combined with CULR will demonstrate significantly greater improvement in UE motor function compared to those receiving sham c-tDCS with CULR. (2) Active c-tDCS will induce greater normalization of cortical hemodynamics, as measured by fNIRS, characterized by a shift in the laterality index. (3) Active c-tDCS will modulate corticospinal excitability, leading to increased MEP amplitudes from the ipsilesional M1 and a more balanced interhemispheric MEP ratio.

## Methods and design

### Study design

**Figure 1:**
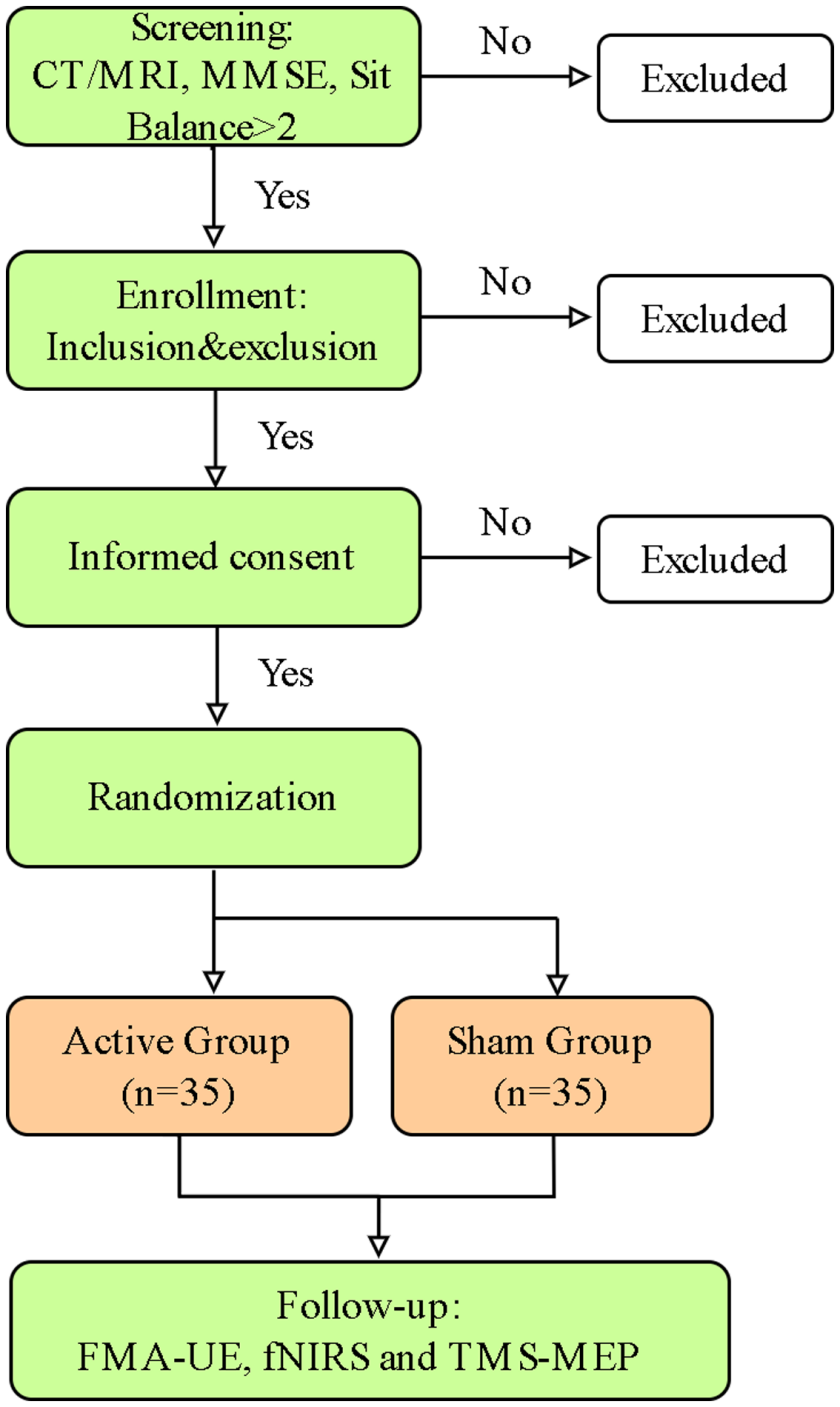
Experimental flow.

The study follows a randomized controlled trial design to examine the efficacy of c-tDCS in improving post-stroke UE motor function and the underlying cortical physiological dynamics will be characterized (Figure 1).

### Study setting

The study is conducted at the Department of Rehabilitation Medicine, Fifth Affiliated Hospital of Guangzhou Medical University in China. Participants are recruited from the inpatient ward, and all interventions are delivered within the department by the hospital staff.

### Recruitment

Patients admitted to the inpatient ward will receive eligibility screening during regular assessment. The clinical team identifies suitable candidates and provides them with written study information. A member of the research team will then approach potential participants, explains the study in detail, and obtains written informed consent from those willing to join. A screening log is maintained to document non-recruited patients and their reasons for exclusion.

### Ethics

Data collection will be performed according to the World Medical Association Declaration of Helsinki. The study has received ethical approval from the Ethics Committee of the Fifth Affiliated Hospital, Guangzhou Medical University (GYWY-L2025-49). Additionally, the study is registered in the Chinese Clinical Trial Registry (Registration No.: ChiCTR2500101094, registered on 21 April 2025). Any significant modifications to the study protocol will be communicated to all relevant parties.

### Participants

#### Sample size

Based on an F-test (ANOVA) with the following parameters: a conservative effect size [*f* = 0.4, based on a meta-analysis of tDCS studies for post-stroke UE recovery (13)], *α* error probability = 0.05, power (1-*β*) = 0.95, numerator df = 1, number of groups = 2, the total sample size is calculated in G-power. Accounting for an anticipated 20% attrition rate, the final required sample size is 70 participants, with 35 in each of the two groups.

**Experimental group:** Patients who receive active tDCS for 10 consecutive days with 20 min each day.
**Control group:** Patients who receive sham tDCS.

#### Randomization and blinding

This study will adhere to the CONSORT guidelines for randomized controlled trials (14). A computer-generated block randomization sequence (with varying block sizes) will be created by an independent statistician not involved in participant recruitment or assessment. The allocation sequence will be concealed in sequentially numbered, opaque, sealed envelopes. After obtaining baseline assessments (T0), a research assistant not involved in the study will open the next envelope to assign the participant to either the active or sham group.

This is a double-blind trial. Participants, the outcome assessors (who perform the FMA-UE, fNIRS, and TMS assessments), and the occupational therapists delivering the conventional rehabilitation will be blinded to group assignment. The investigator responsible for setting up and initiating the tDCS device will not be blinded, as they must input the correct stimulation code (active/sham) for the device. However, this investigator will have no role in outcome assessment or data analysis.

#### Experimental procedure

Eligible participants with unilateral upper extremity motor impairment will be formally screened by a qualified rehabilitation physician according to the predefined inclusion/exclusion criteria (Table 1). Participants will be assigned randomly to the active or sham tDCS group after written informed consent is obtained. The detailed experimental workflow is outlined in Figure 1.

**Table 1:** Inclusion and exclusion criteria.

Inclusion criteria	Exclusion criteria
1. Diagnosis of a first-ever, unilateral, supratentorial ischemic or hemorrhagic stroke, confirmed by CT or MRI;	1. Current pregnancy, lactation or gestation
2. Post-stroke chronicity ranging from 1 week to 2 years;	2. Concurrent participation in clinical trials within the preceding 3 months
3. Ability to maintain assisted sitting posture for ≥40 min without distress;	3. Any clinically significant condition or circumstance deemed by the Principal Investigator to compromise participant safety or protocol adherence at any study phase.
4. MMSE>21	
5. Moderate to severe upper limb motor impairment, defined as a baseline Fugl-Meyer Assessment for the Upper Extremity (FMA-UE) score between 0 and 40	

**Figure 2:**
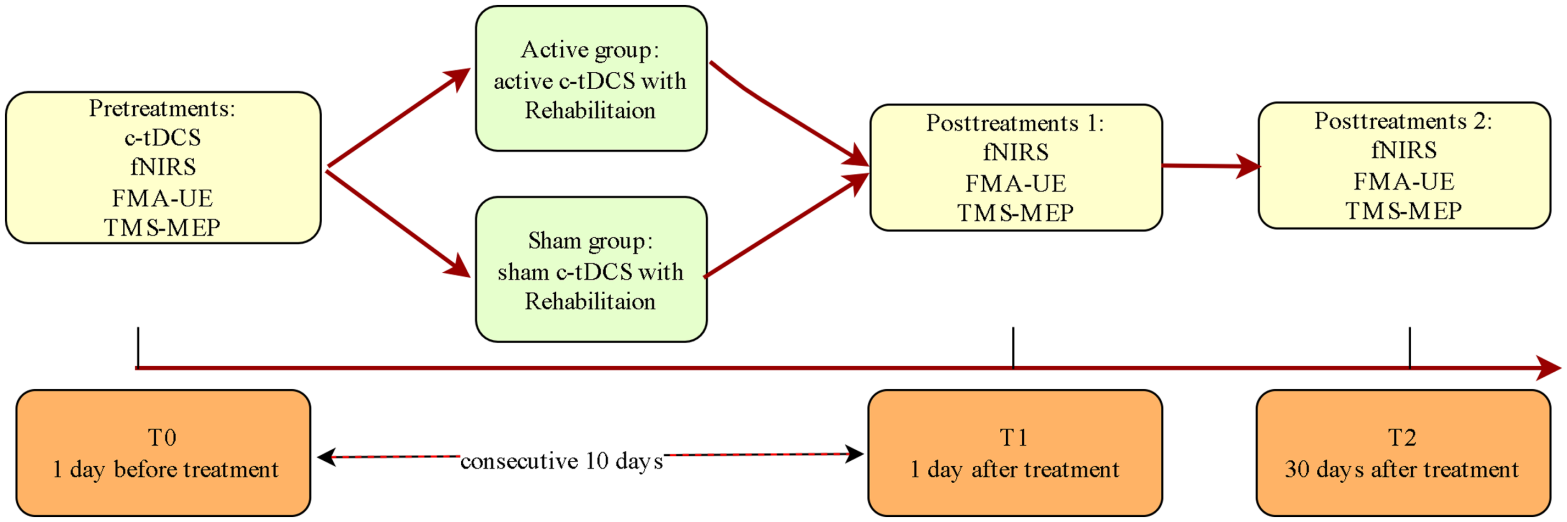
Assessments and interventions.

Complete assessments will be performed at T0, T1, and T2 for both groups (Figure 2 and Table 2).

**Table 2:** Schedule of assessments and outcome measures.

Domain	Outcome measure	Specific variables	Timepoint
Primary	FMA-UE	Total score (0–66)	T0, T1, T2
Secondary	fNIRS	HbO/HbR concentration, Laterality Index (LI)	T0, T1, T2
	TMS-MEP	RMT, MEP Amplitude, MEP Latency, Interhemispheric Ratio	T0, T1, T2
Other	Demographics and clinical data	Age, sex, stroke type, lesion location, time since stroke, etc.	T0

#### fNIRS data acquisition and analysis

**Figure 3:**
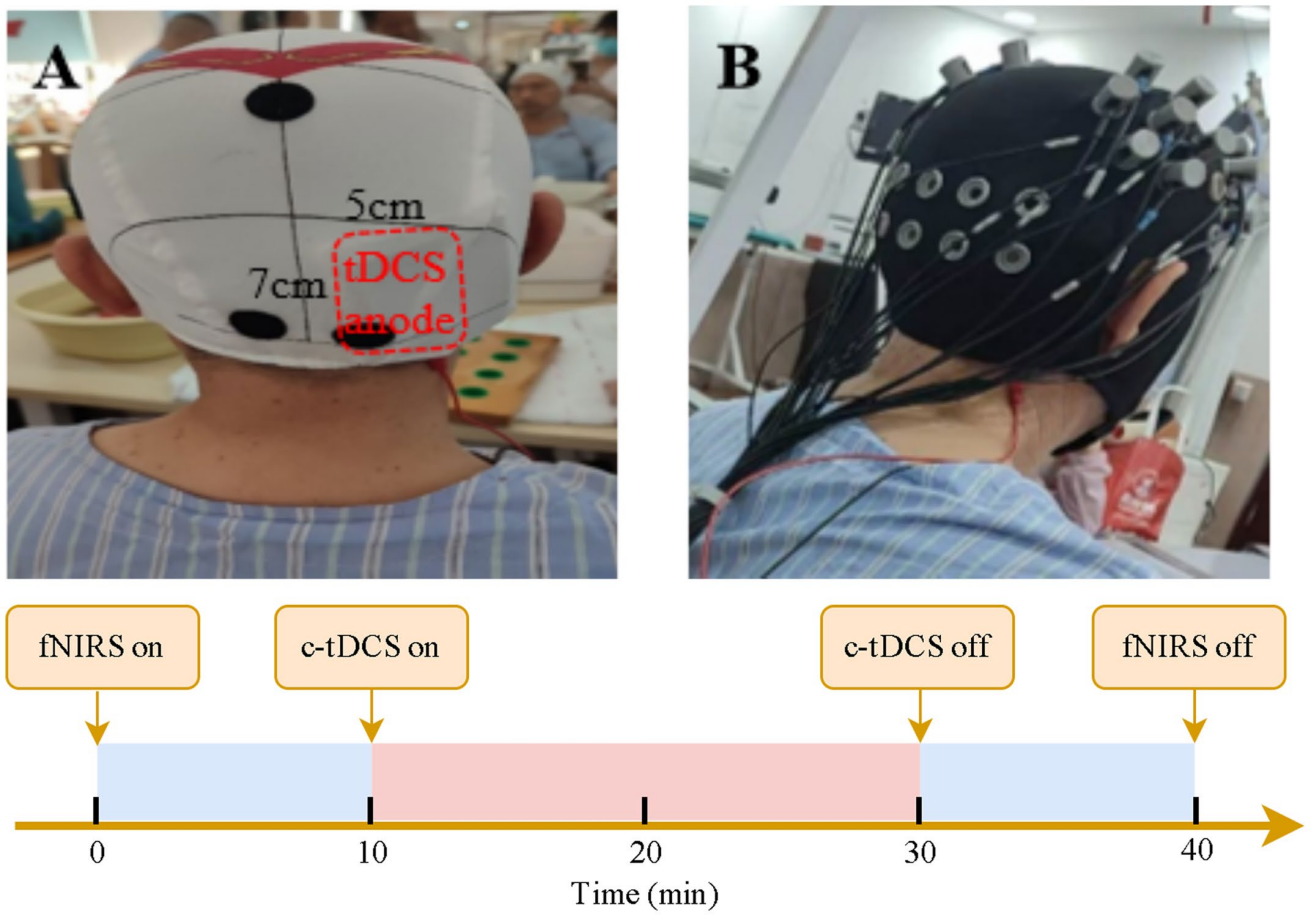
Combined c-tDCS and fNIRS recording at T0. **(A)** depicts the location of anode. **(B)** shows the settings for c-tDCS and fNIRS recording.

The fNIRS data is collected at 3 time points: pre-intervention (T0), post-intervention (T1), and one-month follow-up (T2). Participants will be asked to sit comfortably on chairs in a quiet room, avoiding external interference and instructed to keep still and relax during data collecting period. and relax. The first collection session is conducted before intervention. The fNIRS is continuously running before, during and after tDCS for 40 min in total (Figure 3). The fNIRS sessions at T1 and T2 is recorded in a resting state for 20 min without c-tDCS.

**Figure 4:**
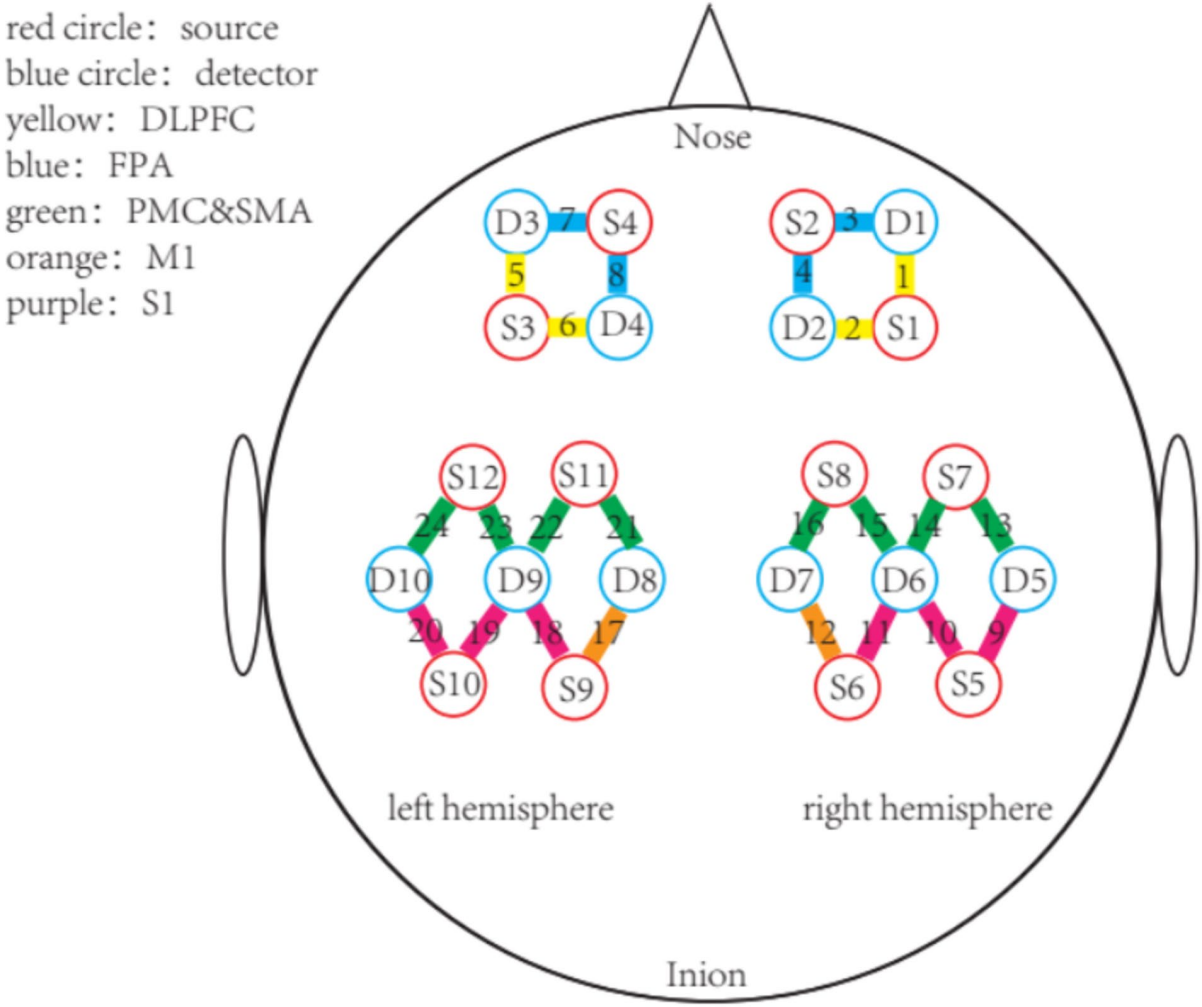
Schematic of the custom fNIRS cap design showing 24 channels covering prefrontal and sensorimotor cortices.

The fNIRS data will be acquired using the NirSmart system (Danyang Huichuang Medical Equipment Co., Ltd., Jiangsu, China). This system continuously monitored concentration changes of oxygenated hemoglobin (HbO) and deoxygenated hemoglobin (HbR) in the cerebral cortex. The apparatus comprised dual-wavelength near-infrared light sources (730 nm and 850 nm light-emitting diodes, LEDs) and avalanche photodiodes (APDs) as detection elements. A configuration of 12 source optodes and 10 detector optodes generated 24 measurement channels. Source-detector separation averaged 3.0 cm (range: 2.7–3.3 cm) across all channels, with hemodynamic data sampled at 11 Hz (Figure 4). Channel coordinates and corresponding brain region calibrations are detailed in Supplementary Table 1.

This study will focus on hemodynamic monitoring within the prefrontal cortex (PFC) and parietal cortex. The data preprocess includes eliminating irrelevant time intervals and motor artifacts, converting the light intensity to optical density, converting the optical density to blood oxygen concentration. The beta value of each ROI will be calculated by general linear model (GLM) in NirSpark software to reflect the hemodynamic response. The concentration of HbO and HbR is calculate based on the Beer–Lambert law and exported to MATLAB R2018a (MathWorks, USA) for further lateralization analysis.

Lateralization Index = (WA affected - WA unaffected)/(WA affected + WA unaffected).

Among the above formulate, the affected wavelet amplitude (WA) is the average WA value of the cortical regions in the lesional hemisphere, while the unaffected WA is the average WA value of the corresponding cortical regions in the contralesional hemisphere. The value range of the WA side degree is −1 to 1. If it is positive, it indicates that the cortical activity on the lesional side is greater than that on the contralesional side; if it is negative, it indicates that the activity on the contralesional side is greater than that on the lesional side.

#### TMS-MEP data measurement and analysis

The TMS is used to assess excitability of bilateral M1 via MEPs. The hand area of both contralesional and ipsilesional M1 will be stimulated using a figure-of-eight coil (70 mm) connected to magnetic stimulators via a BiStim module (NS1000, Yiruide Group, China). The abductor pollicis brevis (APB) cortical hotspot will be operationally defined as the scalp position eliciting MEPs≥50 μV peak-to-peak amplitude in 5 of 10 consecutive stimuli at minimal stimulator intensity. This site will be marked with a surgical pen and retained for follow-up sessions. Resting motor threshold (RMT) will be determined as the minimum intensity required to evoke MEPs >50 μV in 5/10 trials during complete muscle relaxation for both hemispheres. Subsequently, ten MEPs will be recorded at 120% of the RMT from both the contralesional and ipsilesional M1 while the APB is at rest. The peak-to-peak amplitude (in microvolts) and latency (in milliseconds) of these MEPs will be averaged for analysis. The interhemispheric ratio of MEP amplitude (ipsilesional/contralesional) will be calculated as a measure of interhemispheric balance.

#### Interventions

In this study, conventional rehabilitation training is combined either with active tDCS in experimental group or sham tDCS in the control group. The experimental group receive c-tDCS for 10 days, with 20 min per day, while the control group receive pseudo-stimulation of the same duration. The ipsilesional cerebellar hemisphere is stimulated by the anodal tDCS with current intensity of 2 mA. The anode is placed vertically 3 cm lateral to the middle line at the inion level. The ipsilesional cerebellar hemisphere is stimulated by the anodal tDCS with a current intensity of 2 mA. The anode (5 cm×7 cm) is placed vertically 3 cm lateral to the inion. The cathode (5 cm ×7 cm) will be placed over the ipsilateral upper trapezius. In the control group, there is a gradual increase and decrease process of the current at the beginning and end, imitating the scalp discomfort when the current is initiated to ensure the accuracy of the blinding method.

The CULR of the paretic upper extremity includes electrical neuromuscular stimulation, active or passive range of motion training, and muscle strength training. Each CULR session will last for 45 min, delivered by a professional occupational therapist immediately after each tDCS session. The session will be structured as follows: ~15 min of neuromuscular electrical stimulation and passive/active-assisted range of motion exercises for the shoulder, elbow, wrist, and fingers; ~20 min of task-oriented training (e.g., reaching, grasping, manipulating objects of different sizes and weights); and ~10 min of muscle strengthening exercises using resistance bands or light weights. Both groups will receive the same standardized CULR procedure.

## Outcomes

### Primary outcomes

The FMA-UE is a standardized clinical evaluation tool focusing on motor function of the upper extremity (15), comprising 33 assessment items. This scale employs a 3-point scoring criterion (0 = cannot perform; 1 = partially performs; 2 = fully performs), with a total score range of 0–66, where higher scores indicate better motor function (16). Previous studies have demonstrated its excellent validity and reliability (16), establishing it as a robust instrument for assessing post-stroke upper extremity motor impairment. The application of FMA-UE in this study aims to evaluate the clinical efficacy of tDCS targeting the cerebellum in improving upper extremity function after stroke. A change of ≥ 4.25–5.25 points on the FMA-UE will be considered the minimal clinically important difference (MCID) for interpreting the results, based on established values for the stroke population.

### Secondary outcomes

The fNIRS of the cerebral cortex: This study employs a portable 24-channel fNIRS system to noninvasively monitor cortical activity. Key measured parameters include the concentration changes of HbO and HbR. Signal processing involves continuous wavelet transform (CWT) of the HbO to compute WA within the 0.01–0.08 Hz frequency band across cortical regions (17). The inter-hemispheric laterality index (LI) will be then calculated based on WA to evaluate the degree of brain activation transfer from the affected side to the healthy side. WA-derived LI will be calculated based on the HbO concentration pre- and post-intervention by the same computing method.

The TMS-MEP Assessment: TMS is a standardized neurophysiological technique for evaluating the excitability of M1 (18). In this study, RMT is first determined, defined as the minimum stimulation intensity required to elicit MEPs with a peak-to-peak amplitude ≥50 μV in at least five out of ten consecutive trials while the target muscle remains completely relaxed.

Overall, this study employs a multimodal neurophysiological approach combining fNIRS for assessment of cortical hemodynamic responses and TMS-MEP quantitative evaluation of cortical excitability. This integrative methodology provides compelling evidence to elucidate the neuromodulatory mechanisms underlying c-tDCS-induced upper extremity UE motor function changes following stroke (Table 2).

### Data management

Following data management protocols will be strictly adhered to. Firstly, all subject research data will be assigned to unique codes and recorded in case report forms to protect the confidentiality of the participants. Original documents including informed consent forms, medical records, research records, and routine laboratory reports will be archived by the medical institution. These records will be maintained for a minimum of 10 years following study completion, with electronic archiving permitted when security requirements is satisfied. Hospitalization records during the treatment period will be preserved by the research investigator in accordance with standard medical record retention policies. Monitoring of study conduct and data collection will be performed by the committee of the University Clinical Trial Unit on an annual basis. The raw data collected during this research study will be securely stored for a period of up to 5 years after the publication of the main results paper.

### Statistical analysis

The primary analysis will follow the Intention-to-Treat (ITT) principle, including all randomized participants in the groups to which they were originally assigned. For the primary outcome (FMA-UE), missing data will be handled using multiple imputation. A per-protocol (PP) analysis will also be conducted as a sensitivity analysis. To test the primary hypothesis, a mixed-model Analysis of Variance (ANOVA) with factors for Group (active, sham) and Time (T0, T1, T2) will be used to test for the crucial Group x Time interaction effect.

Descriptive statistics will be employed to characterize participants’ clinical profiles. Between-group comparisons will be performed using chi-square tests for categorical variables and independent samples t-tests for continuous variables. Prior to parametric testing, data normality and variance homogeneity will be assessed using the Kolmogorov–Smirnov test and Levene test in the SPSS. All statistical analyses will be performed using SPSS software (version 20.0; SPSS Inc., Chicago, IL, USA). A *p*-value <0.05 will be considered as statistically significant. In addition to the primary analysis, exploratory subgroup analyses will be conducted based on baseline impairment severity (FMA-UE score: moderate [20–40] vs. severe [0–19]) to investigate potential differential treatment effects.

## Discussion

### Summary

The recovery of UE function after stroke makes challenge to the clinical routine. The effectiveness and underlying neural mechanisms of rehabilitation therapy for improving UE function of stroke remain elusive. tDCS is a validated and reliable neuromodulating technique that can be used as a intervention device besides routine rehabilitation activities and fNIRS is a compatible neuroimaging technique (19). Multiple studies have shown that the cerebellum is an effective target for tDCS in improving the UE functions of stroke patients (20). However, there is a lack of research on the neurophysiological activity of the cerebral cortex, as detected by fNIRS or TMS-MEPs, to explore the effect of c-tDCS in stroke patients. Based on previous findings from c-tDCS and fNIRS study in healthy adults, which suggest that unilateral c-tDCS modulate the excitability of bilateral M1, it is hypothesized that the reciprocal connections between ipsilesional cerebellum and contralesional cerebral cortex is preserved and can be utilized for c-tDCS. Additionally, considering the imbalanced interhemispheric inhibition, it is hypothesized that MEPs of the APBs differs, including increased MEPs in the ispilesional side and decreased MEPs in the healthy side.

This study aims to investigate the cortical hemodynamic responses of the cerebral cortex after c-tDCS in stroke patients. In addition, the study aims to explore the UE function recovery and hemodynamic changes in the cerebral cortex brought by the combination of c-tDCS and conventional rehabilitation therapy, therefore to investigate the rehabilitation mechanisms of UE motor function in individuals with stroke.

### Strength and limitations

Our study is one of the first randomized controlled trials to explore the feasibility and effectiveness of combination of ipsilesional anodal c-tDCS and fNIRS in UE function recovery of stroke patients. This study has several strengths. Firstly, it aims to investigate the cortical oxygenation characteristics before, during and after c-tDCS in individuals with stroke. This finding could have the potential to significantly contribute to our understanding of cortical hemodynamic responses to the c-tDCS in stroke patients. It may uncover more valuable insights into the underlying mechanisms of neuromodulation and rehabilitation. Secondly, the utilization of both stimulation and detection system enables stimulating one cortical region and monitoring of blood oxygen parameters in remote regions, providing a comprehensive assessment of cerebellocortical connections. Last but not least, the study employs a combination of cross-setional and interventional design to assess the neuromechanism and effectiveness of the rehabilitation. This comprehensive design enhances the interpretation of the findings, capturing both cross-sectional and longitudinal aspects of stroke patients with UE dysfunction.

There are several important limitations in the present study to be noticed. Firstly, the intervention period is relative short. The short period may not yield significant impact on the functional recovery of UE function. Secondly, the participants will be recruited in a single department from a particular hospital in a specific region of Guangzhou. Thus this could limit the generalization of the final findings. Thirdly, tDCS stimulation intensity is fixed at 2 mA unless it is not bearable for some participants and will be down regulated. The optimal stimulation intensity and duration is not explored in this study.

## Glossary

c-tDCS: Cerebellar Transcranial Direct Current Stimulation
fNIRS: Functional Near-infrared Spectroscopy
FMA-UE: Fugl-Meyer Assessment Upper Extremity
TMS-MEPs: Transcranial Magnetic Stimulation-derived Motor Evoked Potentials
ADL: Activities of daily living
CNS: Central Nervous System
CCD: Crossed Cerebellar Diaschisis
CCP: Corticocerebellar pathway
UL: Upper limb
CT: Computed Tomography
MRI: Magnetic Resonance Imaging
MMSE: Mini-Mental State Examination
ANOVA: Analysis of variance
HbO: Oxygenated hemoglobin
HbR: Deoxygenated hemoglobin
HbT: Total hemoglobin
LEDs: Light-emitting diodes
APDs: Avalanche photodiodes
DLPFC: Dorsolateral prefrontal cortex
PFA: Frontal polar area
PMC: Premotor cortex
SMA: Supplementary motor area
S1: somatosensory cortex
M1: primary motor cortex
ROI: Regions of interest
GLM: General linear model
WA: Wavelet amplitude
APB: Abductor pollicis brevis
RMT: Resting motor threshold
CWT: Continuous wavelet transform
LI: Laterality index
CULR: conventional upper limb rehabilitation
