# Supplementary material for: Effects of cerebellar transcranial direct current stimulation on improving post-stroke upper extremity motor function: a protocol for a randomized controlled clinical trial
Source: Front Neurol. 2025 Nov 18;16:1670721. doi: 10.3389/fneur.2025.1670721 (PMC12669015; doi:10.3389/fneur.2025.1670721)
Supplement: Supplementary file 1 [file Table_1.docx]

**Supplemental table 1**

| Label of SD or CH | Brodmann Area (Chris rorden' MRIcro) | Percentage |
| --- | --- | --- |
| S1 | 45 - pars triangularis Broca's area | 0.3194 |
|  | 46 - Dorsolateral prefrontal cortex | 0.6806 |
|  |  |  |
| S2 | 10 - Frontopolar area | 0.0365 |
|  | 11 - Orbitofrontal area | 0.9336 |
|  | 47 - Inferior prefrontal gyrus | 0.0299 |
|  |  |  |
| S3 | 45 - pars triangularis Broca's area | 0.2258 |
|  | 46 - Dorsolateral prefrontal cortex | 0.7742 |
|  |  |  |
| S4 | 10 - Frontopolar area | 0.0576 |
|  | 11 - Orbitofrontal area | 0.9424 |
|  |  |  |
| S5 | 40 - Supramarginal gyrus part of Wernicke's area | 1 |
|  |  |  |
| S6 | 1 - Primary Somatosensory Cortex | 0.2097 |
|  | 2 - Primary Somatosensory Cortex | 0.176 |
|  | 3 - Primary Somatosensory Cortex | 0.4007 |
|  | 4 - Primary Motor Cortex | 0.1835 |
|  | 7 - Somatosensory Association Cortex | 0.0112 |
|  | 40 - Supramarginal gyrus part of Wernicke's area | 0.0187 |
|  |  |  |
| S7 | 4 - Primary Motor Cortex | 0.004 |
|  | 6 - Pre-Motor and Supplementary Motor Cortex | 0.68 |
|  | 9 - Dorsolateral prefrontal cortex | 0.124 |
|  | 44 - pars opercularis_ part of Broca's area | 0.192 |
|  |  |  |
| S8 | 6 - Pre-Motor and Supplementary Motor Cortex | 0.4719 |
|  | 8 - Includes Frontal eye fields | 0.2734 |
|  | 9 - Dorsolateral prefrontal cortex | 0.2547 |
|  |  |  |
| S9 | 1 - Primary Somatosensory Cortex | 0.2226 |
|  | 2 - Primary Somatosensory Cortex | 0.2566 |
|  | 3 - Primary Somatosensory Cortex | 0.1358 |
|  | 7 - Somatosensory Association Cortex | 0.2377 |
|  | 40 - Supramarginal gyrus part of Wernicke's area | 0.1472 |
|  |  |  |
| S10 | 40 - Supramarginal gyrus part of Wernicke's area | 1 |
|  |  |  |
| S11 | 6 - Pre-Motor and Supplementary Motor Cortex | 0.7836 |
|  | 8 - Includes Frontal eye fields | 0.2015 |
|  | 9 - Dorsolateral prefrontal cortex | 0.0149 |
|  |  |  |
| S12 | 4 - Primary Motor Cortex | 0.0194 |
|  | 6 - Pre-Motor and Supplementary Motor Cortex | 0.7946 |
|  | 9 - Dorsolateral prefrontal cortex | 0.124 |
|  | 44 - pars opercularis_ part of Broca's area | 0.062 |
|  |  |  |
| D1 | 10 - Frontopolar area | 0.0083 |
|  | 11 - Orbitofrontal area | 0.0496 |
|  | 46 - Dorsolateral prefrontal cortex | 0.3719 |
|  | 47 - Inferior prefrontal gyrus | 0.5702 |
|  |  |  |
| D2 | 10 - Frontopolar area | 0.7992 |
|  | 46 - Dorsolateral prefrontal cortex | 0.2008 |
|  |  |  |
| D3 | 10 - Frontopolar area | 0.0804 |
|  | 46 - Dorsolateral prefrontal cortex | 0.5025 |
|  | 47 - Inferior prefrontal gyrus | 0.4171 |
|  |  |  |
| D4 | 10 - Frontopolar area | 0.9576 |
|  | 46 - Dorsolateral prefrontal cortex | 0.0424 |
|  |  |  |
| D5 | 1 - Primary Somatosensory Cortex | 0.2208 |
|  | 2 - Primary Somatosensory Cortex | 0.4416 |
|  | 3 - Primary Somatosensory Cortex | 0.0032 |
|  | 43 - Subcentral area | 0.3344 |
|  |  |  |
| D6 | 3 - Primary Somatosensory Cortex | 0.3254 |
|  | 4 - Primary Motor Cortex | 0.5317 |
|  | 6 - Pre-Motor and Supplementary Motor Cortex | 0.1429 |
|  |  |  |
| D7 | 4 - Primary Motor Cortex | 0.0636 |
|  | 6 - Pre-Motor and Supplementary Motor Cortex | 0.9364 |
|  |  |  |
| D8 | 4 - Primary Motor Cortex | 0.3 |
|  | 6 - Pre-Motor and Supplementary Motor Cortex | 0.7 |
|  |  |  |
| D9 | 1 - Primary Somatosensory Cortex | 0.0987 |
|  | 3 - Primary Somatosensory Cortex | 0.5451 |
|  | 4 - Primary Motor Cortex | 0.3562 |
|  |  |  |
| D10 | 1 - Primary Somatosensory Cortex | 0.3276 |
|  | 2 - Primary Somatosensory Cortex | 0.5358 |
|  | 3 - Primary Somatosensory Cortex | 0.0034 |
|  | 43 - Subcentral area | 0.1297 |
|  | 48 - Retrosubicular area | 0.0034 |
|  |  |  |
| CH1 (S1-D1) | 10 - Frontopolar area | 0.0868 |
|  | 46 - Dorsolateral prefrontal cortex | 0.9132 |
|  |  |  |
| CH2 (S1-D2) | 10 - Frontopolar area | 0.3266 |
|  | 46 - Dorsolateral prefrontal cortex | 0.6734 |
|  |  |  |
| CH3 (S2-D1) | 10 - Frontopolar area | 0.184 |
|  | 11 - Orbitofrontal area | 0.4 |
|  | 46 - Dorsolateral prefrontal cortex | 0.084 |
|  | 47 - Inferior prefrontal gyrus | 0.332 |
|  |  |  |
| CH4 (S2-D2) | 10 - Frontopolar area | 0.7167 |
|  | 11 - Orbitofrontal area | 0.2833 |
|  |  |  |
| CH5 (S3-D3) | 10 - Frontopolar area | 0.2112 |
|  | 46 - Dorsolateral prefrontal cortex | 0.7888 |
|  |  |  |
| CH6 (S3-D4) | 10 - Frontopolar area | 0.3622 |
|  | 46 - Dorsolateral prefrontal cortex | 0.6378 |
|  |  |  |
| CH7 (S4-D3) | 10 - Frontopolar area | 0.228 |
|  | 11 - Orbitofrontal area | 0.556 |
|  | 46 - Dorsolateral prefrontal cortex | 0.06 |
|  | 47 - Inferior prefrontal gyrus | 0.156 |
|  |  |  |
| CH8 (S4-D4) | 10 - Frontopolar area | 0.7379 |
|  | 11 - Orbitofrontal area | 0.2621 |
|  |  |  |
| CH9 (S5-D5) | 1 - Primary Somatosensory Cortex | 0.2244 |
|  | 2 - Primary Somatosensory Cortex | 0.3814 |
|  | 40 - Supramarginal gyrus part of Wernicke's area | 0.3942 |
|  |  |  |
| CH10 (S5-D6) | 1 - Primary Somatosensory Cortex | 0.5423 |
|  | 2 - Primary Somatosensory Cortex | 0.0115 |
|  | 3 - Primary Somatosensory Cortex | 0.2654 |
|  | 40 - Supramarginal gyrus part of Wernicke's area | 0.1808 |
|  |  |  |
| CH11 (S6-D6) | 1 - Primary Somatosensory Cortex | 0.166 |
|  | 3 - Primary Somatosensory Cortex | 0.502 |
|  | 4 - Primary Motor Cortex | 0.332 |
|  |  |  |
| CH12 (S6-D7) | 3 - Primary Somatosensory Cortex | 0.0537 |
|  | 4 - Primary Motor Cortex | 0.7416 |
|  | 6 - Pre-Motor and Supplementary Motor Cortex | 0.2047 |
|  |  |  |
| CH13 (S7-D5) | 1 - Primary Somatosensory Cortex | 0.0105 |
|  | 3 - Primary Somatosensory Cortex | 0.1014 |
|  | 4 - Primary Motor Cortex | 0.2413 |
|  | 6 - Pre-Motor and Supplementary Motor Cortex | 0.3112 |
|  | 43 - Subcentral area | 0.3357 |
|  |  |  |
| CH14 (S7-D6) | 4 - Primary Motor Cortex | 0.1474 |
|  | 6 - Pre-Motor and Supplementary Motor Cortex | 0.8526 |
|  |  |  |
| CH15 (S8-D6) | 4 - Primary Motor Cortex | 0.1654 |
|  | 6 - Pre-Motor and Supplementary Motor Cortex | 0.8346 |
|  |  |  |
| CH16 (S8-D7) | 6 - Pre-Motor and Supplementary Motor Cortex | 1 |
|  |  |  |
| CH17 (S9-D8) | 1 - Primary Somatosensory Cortex | 0.024 |
|  | 3 - Primary Somatosensory Cortex | 0.2979 |
|  | 4 - Primary Motor Cortex | 0.661 |
|  | 6 - Pre-Motor and Supplementary Motor Cortex | 0.0171 |
|  |  |  |
| CH18 (S9-D9) | 1 - Primary Somatosensory Cortex | 0.3147 |
|  | 2 - Primary Somatosensory Cortex | 0.1235 |
|  | 3 - Primary Somatosensory Cortex | 0.4104 |
|  | 4 - Primary Motor Cortex | 0.1514 |
|  |  |  |
| CH19 (S10-D9) | 1 - Primary Somatosensory Cortex | 0.3765 |
|  | 2 - Primary Somatosensory Cortex | 0.098 |
|  | 3 - Primary Somatosensory Cortex | 0.2314 |
|  | 40 - Supramarginal gyrus part of Wernicke's area | 0.2941 |
|  |  |  |
| CH20 (S10-D10) | 1 - Primary Somatosensory Cortex | 0.0507 |
|  | 2 - Primary Somatosensory Cortex | 0.3732 |
|  | 40 - Supramarginal gyrus part of Wernicke's area | 0.5761 |
|  |  |  |
| CH21 (S11-D8) | 4 - Primary Motor Cortex | 0.0109 |
|  | 6 - Pre-Motor and Supplementary Motor Cortex | 0.9891 |
|  |  |  |
| CH22 (S11-D9) | 4 - Primary Motor Cortex | 0.3128 |
|  | 6 - Pre-Motor and Supplementary Motor Cortex | 0.6872 |
|  |  |  |
| CH23 (S12-D9) | 3 - Primary Somatosensory Cortex | 0.004 |
|  | 4 - Primary Motor Cortex | 0.3992 |
|  | 6 - Pre-Motor and Supplementary Motor Cortex | 0.5968 |
|  |  |  |
| CH24 (S12-D10) | 1 - Primary Somatosensory Cortex | 0.1206 |
|  | 3 - Primary Somatosensory Cortex | 0.1738 |
|  | 4 - Primary Motor Cortex | 0.2979 |
|  | 6 - Pre-Motor and Supplementary Motor Cortex | 0.1879 |
|  | 43 - Subcentral area | 0.2199 |
